# *Rubus Fruticosus* L.: Constituents, Biological Activities and Health Related Uses

**DOI:** 10.3390/molecules190810998

**Published:** 2014-07-28

**Authors:** Muhammad Zia-Ul-Haq, Muhammad Riaz, Vincenzo De Feo, Hawa Z. E. Jaafar, Marius Moga

**Affiliations:** 1 The Patent Office, Kandawala Building, M.A. Jinnah Road, Karachi-74400, Pakistan; 2 Department of Pharmacy, Shaheed Benazir Bhutto University, Sheringal, Dir Upper-2500, Pakistan; E-Mail: pharmariaz@gmail.com; 3 Department of Pharmaceutical and Biomedical Sciences, University of Salerno, Salerno 84100, Italy; E-Mail: defeo@unisa.it; 4 Department of Crop Science, Faculty of Agriculture, University Putra Malaysia, Selangor, 43400, Malaysia; E-Mail: hawazej@agri.upm.edu.my; 5 Department of Medicine, Transilvania University of Brasov, Brasov 500036 Romania; E-Mail: moga.og@gmail.com

**Keywords:** *Rubus fruticosus* L., pharmacology, phytochemistry, nutrition

## Abstract

*Rubus fruticosus* L. is a shrub famous for its fruit called blackberry fruit or more commonly blackberry. The fruit has medicinal, cosmetic and nutritive value. It is a concentrated source of valuable nutrients, as well as bioactive constituents of therapeutic interest highlighting its importance as a functional food. Besides use as a fresh fruit, it is also used as ingredient in cooked dishes, salads and bakery products like jams, snacks, desserts, and fruit preserves. *R. fruticosus* contains vitamins, steroids and lipids in seed oil and minerals, flavonoids, glycosides, terpenes, acids and tannins in aerial parts that possess diverse pharmacological activities such as antioxidant, anti-carcinogenic, anti-inflammatory, antimicrobial anti-diabetic, anti-diarrheal, and antiviral. Various agrogeoclimatological factors like cultivar, environmental conditions of the area, agronomic practices employed, harvest time, post-harvest storage and processing techniques all influence the nutritional composition of blackberry fruit. This review focuses on the nutrients and chemical constituents as well as medicinal properties of different parts of *R. fruticosus*. Various cultivars and their physicochemical characteristics, polyphenolic content and ascorbic acid content are also discussed. The information in the present work will serve as baseline data and may lead to new biomedical applications of *R. fruticosus* as functional food.

## 1. Introduction

Plant foods (fruits, herbs, nuts, spices, vegetables, legumes and grains) occupy an important position in the economic, cultural as well as health systems of both developing and developed countries due to their proven health-promoting claims and immunity-boosting effects. Regular consumption of fruits, spices, nuts, legumes, vegetables and grains, is vital for a balanced and nutritious diet and is associated with reduced risk of various ailments like inflammation, arthritis, cancer, diabetes, cardiovascular disease, atherosclerosis, cataracts, Parkinson’s disease, Alzheimer’s disease, and aging. The origin of many remedies, recipes and pharmaceuticals can be been plant food especially fruits. Nutritional information of fruits and their effects on human health is among the most frequently referenced and most sought-after items on the internet. Fruits are consumed in various quantities as concentrated sources of energy, nutrition, vitamins, essential minerals and antioxidants by people of all ages and income groups globally. *Rubus fruticosus* L. (*Rosaceae*) is a shrub famous for its fruit, called blackberry, which is traded globally due to its delicious taste, pleasant flavor and nutritional profile. The shrub is believed to have its origin in Armenia, and is now distributed throughout Europe, Asia, Oceania and North and South America [1,2,3]. It grows wild in the Northern areas of Pakistan, like Chitral [4], Dir [5], Mansehra [6], Malakand [7,8] and Kotli [9], where it is known by local names *Karwara* [4,7], *Ach* [8], *Akhara* [6] and *Baganrra* [10,11]. Although the fruit has wide acceptance in Pakistan, it is not cultivated on a commercial scale. The *Rosaceae* family is the 19th largest family of plants [12]. The genus *Rubus*, with almost 700 species, is the largest genus of this family [13]. *Rubus* comprises 12 subgenera, with few domesticated species [14]. Members of this genus have been cultivated for centuries for their fruits. These fruits are consumed fresh or processed to make food products such as jam, wine, tea, ice cream, desserts, seedless jellies and bakery products. Extracted pigment from fruits is used as a natural colorant in baked products, jellies, chewing gums, fruit-wines and beverages [15,16]. Due to increasing awareness about the valuable attributes of functional foods and optimal nutrition among customers, the global consumption of fruits and fruit-based products has increased considerably, especially in high-income countries. It is well-known now that healthiest diets are those loaded with plant foods, especially fruit-based diets. Therefore, health care advisors and nutrition counselors recommend inclusion of fruits and fruit-based products especially juices in the diet. Blackberries possess a delicious taste, pleasant flavor, nice appearance and excellent nutritional profile. Fruit are eaten raw or cooked as well as crushed to make juice. Syrups, jams and other preserves are prepared from fruit [17]. The cooked root is also used as food [18], while leaves, whither dried or fresh, are used as a tea [19]. The young shoots are used in salads after peeling [20].

The present work is a survey of research carried out on this plant. Various search engines like SciFinder, PubMed and ScienceDirect were used to search the isolated bioactive constituents and pharmacological activities exhibited by these compounds as well as by the crude extracts by using the search-terms *Rubus fruticosus*, chemical constituents and pharmacological activities as keywords. The main objective of the present review is to compile a comprehensive report covering medicinal, phytochemical and nutritional attributes of different parts of the blackberry.

## 2. Botanical Description

### 2.1. Description

*Rubus fruticosus* L. is a semi-prostrate to almost erect, scrambling, perennial deciduous prickly, shrub with entangling and arching stem growing up to 3 m at a fast rate. It grows in woodland garden sunny edge, dappled shade, shady edges [21]. This bushy plant is thorny, but some cultivated varieties are free of thorns. Blackberries are perennial, lasting for three seasons or more [3]. Plants typically bear biennial stems or semi woody called canes. They vary from sprawling to almost erect, spreading shrubs with thorn and leaves, the stem grow up to 7 m in length that is greenish, purplish or red in colour. Every spring buds of the woody root produce juvenile canes which grow at a fast pace of almost 50–80 mm per day [3]. They are categorized into two groups in terms of branch structure: generative cane (floricane) and vegetative cane (primocane). Vegetative canes formed during first year convert into generative canes during the second year [22]. The plant flowers in early summer and late spring. Diameter of a flower is about 2–3 cm having 5 pale pink or white petals. Flowers have multiple stamens. After fall of petals, fruit develops an aggregate of drupelets that are green earlier and later turn to red to black on ripening. The color of fruit and fruit juice is an important parameter from commercial point of view as consumers rate the product depending upon its visual appearance. The color of blackberry fruit and its juice depends upon natural pigments present in it which in turn depends upon many factors like cultivar being analyzed, agronomic practices utilized in cultivation, maturity stage of collection and geological and climatic conditions of area from where fruit is collected, post-harvest storage conditions employed and enzymatic activity and microbial contamination. Juice may be extracted from fresh blackberry as well as from frozen. The color of frozen is much better than fresh one. Flowers and fruit occur in a panicle-like or raceme [3]. They are formed in clusters at the end of floricanes. Blackberry fruits twice a year both in spring (floricane) and autumn (primocane) [22]. A dense cluster of separate units or drupelets forms the fruit which on ripening turn black or dark purple from red [3]. Seeds are light to dark brown in colour, round, 2–3 mm long with irregular and deep pits. The upper side of leaves is dark green while underside is lighter green. Short prickles cover the stalks and veins of leaves. Leaves are ternate above, tending to 5 or 7 palmate leaflets towards the base. Adaxial sides of these leaflets are folded into pleats and glabrate which are dark red-purple in fall, green in summer and deciduous in winter [3].

### 2.2. Cultivars

Numerous cultivars of *R. fruticosus* have been developed by farmers by traditional breeding methods. These cultivars differ in fruit firmness, shape, size, flavor, color, weight, yield, ripening season, nutritional contents and resistance to pests. The most famous cultivars are Jumbo, Chester, Bartin, Ness, Bursa 1, Bursa 2, Bursa 3, Arapaho, Navaho, Thornfree, Chester Thornless, Dirksen Thornless, Cacanska Bestrna, Loch Ness, Cherokee, and Black Satin [23,24].

### 2.3. Physico-Chemical Characteristics of Fruit and Oil

The increasing awareness of consumers about healthy and functional food has led to increased consumption of fruit and fruit-based products. Physico-chemical characteristics of fruit are the key parameters that define quality of fruit and products made there from. A good fruit flavor is due to higher levels of sugar and organic acids. Various parameters of fruit like fruit dimensions, weight, titratable acidity (TAc), pH and total soluble solids (TSS) contents of cultivated and wild blackberry fruits were determined by Yilmaz *et al.* [23]. Fruit weight ranged from 1.2 g to 5.4 g for Arapaho and Bursa1 cultivars respectively while it was 0.4 g to 1.2 g for wild genotypes. It indicates that cultivated genotypes have higher mean fruit weight as compared to wild genotypes; same trend was observed for length and width of fruit. However TSS was less in cultivated genotypes (8.6%–14.1%) than wild genotypes (12.9%–22.3%) with overall means of 11.6% *vs.* 16.2%. The total soluble solid means of wild genotypes was higher by 20%. The pH means of the wild genotypes were slightly but significantly higher than the cultivated genotypes.

**Table 1:** Fruit weight, berry size and berry shape index of blackberry fruits grown in Serbia [22].

Cultivars	Fruit Weight (g)		Length (mm)		Width (mm)		Shape Index	
Year	2010	2011	2010	2011	2010	2011	2010	2011
Cacanska Bestrna	7.57	7.61	26.62	27.54	20.31	21.35	1.31	1.29
Black Satin	6.45	7.24	25.96	27.08	20.40	21.28	1.27	1.27
Thornfree	4.65	5.32	21.52	23.69	17.52	19.31	1.23	1.22
Loch Ness	7.76	7.61	28.13	27.15	21.78	20.69	1.30	1.32
Dirksen Thornless	4.54	6.91	27.28	28.10	19.31	20.33	1.41	1.38
Chester Thornless	5.31	6.11	24.13	25.01	19.72	20.82	1.23	1.20
Navaho	5.39	5.90	22.65	23.12	19.46	19.80	1.16	1.17
Mean over years	5.95	6.67	25.18	25.96	19.78	20.51	1.27	1.26

**Table 2:** Soluble solids, titratable acidity and ripening index of blackberry fruits grown in Serbia [19].

Cultivar	Soluble Solids (°Brix)		Titratable Acidity (%)		Ripening Index	
Year	2010	2011	2010	2011	2010	2011
Thornfree	7.70	8.66	1.72	1.60	4.48	5.41
Cacanska Bestrna	6.40	7.82	1.89	1.64	3.39	4.77
Loch Ness	9.25	9.35	1.56	1.42	5.93	6.58
Dirksen Thornless	6.80	9.76	1.51	1.24	4.50	7.87
Black Satin	6.70	6.89	1.57	1.42	4.27	4.85
Chester Thornless	9.20	9.27	1.44	1.27	6.39	7.30
Navaho	9.35	9.67	1.33	1.08	7.03	8.95
Mean over years	7.91	8.77	1.57	1.38	5.14	6.53

Milosevic *et al.*, compared physio-chemical characteristics of fruits of different cultivars blackberry grown in Serbia in two years. A large variation was observed in parameters investigated. (Table 1 and Table 2) [24].

The soluble solids which represent sugar level in fruits and pH and titratable acids which represent total acids contribute to sweetness and acidity of fruits and products made from them. Blackberry cultivars grown in different regions of Turkey had total soluble solids (8.98%–20.2%), weight (2.0–6.6 g), pH (3.3–3.6) and acidity (1.0%–3.1%) for cultivated blackberry while weight (1.5–2.1 g), TSS (11.3%–13.1%), pH (3.33–3.35) and acidity (0.7%–1.0%) for wild blackberries [25,26,27]. The fruit acidity is due to presence of organic acids especially malic acid. The balance between soluble solids contents and titratable acidity is determined by sugars and organic acids ratio and this determines flavor of fruit. Fruit parameters like fruit dimensions, weight, titratable acidity (TAc), pH and total soluble solids (TSS) contents depends upon fruit variety, agronomic practices employed, stage of collection of fruits and climatic and geological condition of area from where fruits are collected. Determination of these parameters is of main interest and first step during nutritional evaluation of fruits and it dictates further studies on components which seem more interesting. Dimića *et al.*, reported the technological quality characteristics of dried pomace of blackberry as well as total carotenoid and chlorophyll contents and physio-chemical characteristics of oil (Table 3 and Table 4). Fresh fruits were frozen for 8 months and then pressed to extract juice. The residue obtained from pressing fruits (pomace) was collected and dried by two ways:

B1 = pomace dried (22 °C) for 3 days
B2 = pomace dried at 63 ± 2 °C and 103 ± 2 °C for 20 h each

**Table 3:** Technological quality parameters of blackberry seeds grown in Serbia [28].

Parameter	Blackberry Seeds	
	B1	B2
Water content (%)	6.59	5.24
Oil content (%)		
- telquel (as is)	13.05	13.59
- on dry basis	13.97	14.34
Impurities content (%)	4.68	4.36
Pure seeds content (%)	95.32	95.64
Weight of 1000 seeds * (g)		
- telquel (as is)	3.21	3.32
- on dry basis	3.45	3.50
Specific weight (g/mL)		
- pure seeds *	0.999	0.997
- telquel seeds (as is)	0.997	0.993
Weight per liter (g/L):		
- pure seeds *	423.6	429.2
- telquel seeds (as is)	384.8	394.0

*: pure separated seeds from pomace by hand.

**Table 4:** Important quality parameters of blackberry seed oils grown in Serbia [28].

Parameter	Blackberry Seed Oil	
	B1	B2
Acid value (mg KOH/g)	6.85	7.05
FFA (% oleic acid)	3.43	3.53
Peroxide value (mmol/kg)	8.89	11.16
Total carotenoids (mg/kg)	32.30	33.92
*Total chlorophyll (mg/kg)* Cyclohexane	3049.52	3094.98
Chloroform	1505.78	1583.62
*Transparency (%)* Cyclohexane	25.84	19.28
Chloroform	24.33	18.87

A brown-greenish oil (due to presence of high chlorophyll contents) was obtained by *n*-hexane extraction of this pomace. Various physico-chemical parameters of this oil were studied. Total chlorophyll content as well as transparency (%) of oil was studied by dissolving oil in two different solvents (*i.e*., cyclohexane and chloroform) and results were significantly different for both solvents [28].

## 3. Phytochemistry

The profile and contents of bioactive contents and constituents, fixed and essential oil, fatty acids, tocopherol and sterols, minerals, amino acids, vitamins, protein and carbohydrate contents of fruits or products made from them depends upon fruit variety, agronomic practices utilized in cultivation, stage of collection of fruit, geological and climatic conditions of area from where fruit is collected and the method utilized for their determination. Proper identification and quantification of bioactive constituents is necessary to understand the underlying mechanism of biological and pharmacological activities of extracts of plants as these properties are due to presence of bioactive constituents. Blackberry fruit itself, and its products as well as by-products are a rich source of phytochemicals and natural antioxidants which are being explored for their health promoting activities. Detailed quantitative data of bioactive components is still needed and their structure activity relationship should be investigated.

### 3.1. Compositional Studies of Fruit

Due to the proven benefits of regular consumption of fruit and vegetables in promoting health and combating metabolic disorders and chronic diseases like cancer, diabetes mellitus, hypertension, cardiovascular diseases, gastrointestinal diseases, atherosclerosis, aging, Parkinson’s and Alzheimer’s disease in humans, their consumption has increased globally. The health benefits of fruits and fruit products are due to their low calories, less energy density and low fat contents, higher vitamins, minerals, fibre and simple sugar contents and presence of various bioactive constituents in them. The nutritional profile of berry fruit indicates presence of carbohydrates, vitamins, minerals as well as dietary fibre (Table 5).

This profile of fruit indicates its potential use in diet-based therapies for improving human health. Due to high water content, carbohydrate content of fruit is less as compared to cereals. Like other fruits, its fruits also have less quantity of protein and sodium. Usually, protein content of fruits is less than 3.5% with exceptions. Similarly lipid content of its fruits like other fruits is not greater than 1%. Like most other fruits, it is rich in potassium which may help in reducing risk of developing kidney stones, bone loss and blood pressure. Milosevic *et al.*, performed a comparative study of sugar and ascorbic acid contents of fresh fruits of blackberry (Table 6) [29].

**Table 5:** Blackberries nutritive value per 100 g [30].

Component	Nutrient Value	Percentage of RDA
Energy	43 Kcal	2%
Carbohydrates	9.61 g	7%
Total Fat	0.49 g	2%
Protein	1.39 g	2%
Dietary Fiber	5.3 g	14%
Cholesterol	0 mg	0%
Folates	25 µg	6%
Pyridoxine	0.030 mg	2%
Niacin	0.646 mg	4%
Pantothenic acid	0.276 mg	5.5%
Thiamin	0.020 IU	2%
Vitamin C	21 mg	35%
Vitamin A	214 IU	7%
Vitamin K	19.8 µg	16.5%
Vitamin E	1.17 mg	8%
Potassium	162 mg	3%
Calcium	29 mg	3%
Sodium	1 mg	0%
Magnesium	20 mg	5%
Copper	165 µg	18%
Iron	0.62 mg	8%
Zinc	0.53 mg	5%
Manganese	0.646 mg	3%
Selenium	0.4 µg	1%
Carotene-α	0 µg	--
Carotene-β	128 µg	--
Lutein-zeaxanthin	118 µg	--

RDA = Recommended daily allowance.

**Table 6:** Sugar and ascorbic acid contents (FW) in blackberry cultivars grown in Serbia [30].

Cultivars	Reducing Sugars (%)	Sucrose (%)	Total Sugars (%)	Ascorbic Acid (mg /100 g)
Black Satin	5.65	0.98	6.68	38.72
Dirksen Thornless	7.98	1.00	9.04	35.20
Chester Thornless	8.18	0.89	9.12	36.96
Thornfree	6.12	0.86	7.02	40.48
Čačanska Bestrna	7.36	0.85	8.25	42.24
Loch Ness	9.01	0.90	9.96	44.00
Navaho	9.08	1.08	10.22	35.20

Since ascorbic acid is a water-soluble vitamin, it is present in excessive amounts in fruits and vegetables having water contents more than 50%. It explains higher level of ascorbic acid in blackberry fruit. The fruit is a rich source of carbohydrates most of which is present as sugars thereby making fruit a high source of energy. These sugars are also a basis of sweetness of fruit. The fruit may be included in nutritional support and dieto-therapy programs to prevent lifestyle-related diseases like diabetes mellitus and cancer due to presence of sufficient amount of ascorbic acid and folic acid.

Fruits and fruit juices are very important in human nutrition as vital source of nutrients, non-nutritive food constituents and for reduction of various disease risks. Therefore ad commercials and campaigns to increase their consumption are justified as a policy to decrease burden of diseases. Stajcic *et al.*, reported chemical composition of two blackberry cultivars *i.e.*, Cacanska bestrna and Thornfree (Table 7) [31]. The compositional data of fruits is a vital information for food scientists as it is an index of total energy content, nutrients and calories present in that fruit. This information helps to establish the relationships between fruit intake and disease in specific population and also helps in formulation of recommended dietary intakes (RDI) and recommended dietary allowance (RDA) values for that fruit.

**Table 7:** Chemical composition of two blackberry cultivars grown in Serbia [31].

Parameter (g/100 g FW *)	Čačanska Bestrna	Thornfree
Total solids	11.96	15.57
Ash	0.29	0.41
Cellulose	2.2	2.97
Pectin	0.29	0.30
Pectic acid	0.1	0.10
Protopectin	0.15	0.17
Acidity	1.36	1.39
Total sugars	5.36	5.98
Reducing sugars	1.46	1.32
Sucrose	3.71	4.43
Proteins	1.4	1.49

* FW: fresh weight of berry fruits.

Various other scientists also reported glucose [32], fructose [33] and sucrose from the fruit [34]. Pectins have also been reported from the fruit of *R. fruticosus* [35]. Organic acids are primary metabolites found mostly in fruits. Various organic acids like citric [32] malic [33] and galacturonic acids [35] have been found in the fruit. Organic acids are usually present in minor concentration in fruits and are responsible for fruit flavor. They help to stabilize anthocyanins and ascrorbic acid in fruits and these acids in combination with sugar also impart sensory characteristics to fruits. The composition of sugars detected in blackberry (fruits) indicates that fructose is predominant, followed by glucose [36]. Since fructose is sweeter than glucose, its high concentration is a desirable organoleptic characteristic of fruits. In a recent study, blackberry expressed the lowest values of fructose, sucrose and glucose contents (64.5 mg/g FW, 76.1 mg/g FW, 3.0 mg/g FW) respectively. “Thornfree” had highest levels of fructose and glucose. Sucrose was present in much lower quantities as compared to the other sugars in wild varieties, because it is converted to inverted forms during the ripening process. Significant differences in malic acid content were observed between wild and cultivated species [37]. Vitamins such as A, C, E, and folic acid were reported in fruit powder of *R. fruticosus* during anticancer studies on berries [38].

Stefanut *et al.*, reported macro-mineral and micro-mineral concentration of Zn, Cu, Al, Mn, Co, Fe as 140, 50, 27, 33, 1, 30 (μg/100 g fruits) in fresh blackberry (fruit) respectively [39]. Radocaj *et al.*, reported that pomace, even after extended frozen storage, is a good raw material for oil extraction and a rich source of functional bioactive constituents (Table 8). The quality characteristics of blackberry seed oils were studied. The results indicated that prolonged freezing time as well as pomace drying method did not influence fatty acid profile of oils extracted from pomaces. The results indicated that best drying regime for blackberry pomace was the two step drying process [40]. Presence of higher amounts of α-tocopherol in pomace and its known highest biological activities than other tocopherols, indicates potential use of pomace in food, pharmaceutical and cosmetics industries as value added natural extract.

**Table 8:** Chemical composition of oils extracted from blackberry pomaces grown in Serbia [40].

Parameter	B0	B1	B2
Water content (%)	6.08	6.55	5.20
FFA (% oleic acid)	1.18	3.44	3.54
PV (mmol/kg)	3.73	8.84	11.14
Induction period (h) at 100 °C	7.50	6.30	6.80
Campesterol	781.7	757.1	771.8
Stigmasterol	1090.4	1052.9	1087.1
β-sitosterol	4370.5	4331.9	4337.9
Total sterols content (mg/kg)	6242.6	6159.8	6196.8
α-tocopherol	133.2	79.1	110.7
β-tocopherol	1097.9	1051.9	1062.5
γ-tocopherol	823.2	565.7	624.5
Total tocopherols (mg/kg)	2054.3	1696.7	1797.7
Σ-SFA	7.53	7.13	7.48
Σ-MUFA	19.97	17.87	19.03
Σ-PUFA	78.56	74.94	75.66
Total phenolics content (mg GAE/kg)	306.5	226.9	256.6

B0: fresh dried at 22 °C/72 h; B1: freeze dried at 22 °C/72 h; B2: freeze dried at 63 °C/20 h and 103 °C /2 h.

α-tocopherol, γ-tocopherol, δ-tocopherol and γ-tocotrienol were reported in seed oils from Korean thornless blackberry [41,42,43]. Mazur and co-workers isolated Δ^7^-avenasterol, squalene, daidzein, genistein, secoisolariciresinol and matairesinol from fruits of *R. fruticosus*. Other sterols in the seed oil of *R. fruticosus* include campesterol, Δ^5^-avenasterol, stigmasterol and β-sitosterol [44]. Both saturated and unsaturated fatty acids have been observed in seed oil, the major fatty acids being lauric, myristic, palmitic, stearic, oleic, linoleic, α-linolenic and arachidic acids. Lead was detected in shoots and roots [45] while rare earth elements, viz La, Lu, Ce, Yb, Sm, Tb, Nd and Eu, were found in leaves of *R. fruticosus* [46]. Toth and coworkers reported the presence of minerals, viz chromium, zinc, manganese, calcium, copper, iron and nickel, in fruit and leaves of *R. fruticosus* [47].

### 3.2. Phenolic Acids, Flavonoids and Anthocyanins

The health promoting properties and immunity-boosting effects of fruits, vegetables and products made from them depend on concentration and profile of phenolic acids, flavonoids, carotenoids, anthocyanians, vitamins and minerals present in them as well as on quantity and frequency of their daily intake and their bio-avilibity to human physiological system after digestion. Therefore determination of phenolic acids, flavonoids, carotenoids, anthocyanians, vitamins and minerals is of prime importance in assessment of nautraceutical values. Total phenolic contents determined in a recent study [31] are from 1.74 mg GAE/g to 1.97 mg GAE/g which are in good agreement with previously published data [48]. These results are slightly lower than those obtained in some thornless blackberry cultivars grown in Italy [49]. Phenolic acids, like ellagic, gallic, caffeic acid and *p*-coumaric acids, and flavonoids, such as quercetin, hyperoside, kaempferol, myricitin, (+)-catechin, (–)-epicatechin, epicatechin gallate, procyanidin B1 and quercetin-3-d-glucoside, have been identified in fruit and leaves of *R. fruticosus* [34,50,51,52,53,54,55]. Radovanović *et al.* [50] has reported individual contents of phenolic acids present in blackberry fruit (Table 9).

**Table 9:** Phenolic acids profile of blackberry fruit [50].

Phenolic Acid	Contents (mg/kg Fresh Weight)
Gallic acid	137.98
*t*-Caftaric acid	0.99
Caffeic acid	0.33
Syringic acid	3.71
Procyanidin B2	1.49
(+)-Catechin	4.09
(‒)-Epicatechin	3.63
Quercetin-3-Glycoside	3.53
Rutin	22.77
Quercetin	3.79

Sellappan and co-workers compared the chemical composition of wild blackberry and common commercial cultivars for ellagic acid and flavonols. They found significant differences between the amounts of individual flavonols and ellagic acid. Wild cultivars of *R. fruticosus* contained the highest values of myricetin, kaempferol, and ellagic acid contents. Wild cultivars of *R. fruticosus* had twice higher contents of ellagic acid as compared to cultivated genotypes [56].

Anthocyanins are a group of flavonoid derivatives and water soluble natural pigments as they give color to flowers and fruits. Animal model studies indicate that anthocyanins possess anti-carcinogenic, anti-inflammatory, and anti-obesity activities besides their role in preventing diabetes mellitus and cardiovascular diseases.The total anthocyanin content of a blackberry crude extract was 17.1 mg/g of freeze-dried powder, which was equival to 176 mg/100 g of blackberry [57]. The primary anthocyanin detected in blackberry is cyanidin-3-O-glucoside. Various other anthocyanins are also detected in blackberry fruit like cyanidin-3-O-xyloside, cyanidin-3-O-dioxaloylglucoside and cyanidin-3-O-(600-malonyl)-glucoside [58]. Smaller amounts of other anthocyanins reported in blackberry are pelargonidin-3-O-glucoside, malvidin-3-O-glucoside, cyanidin-3-O-arabinoside, cyanidin-3-O-xyloside, cyanidin-3-O-rutinoside, cyanidin-3-O-dioxalylglucoside and cyanidin-3-O-glucoside acylated with malonic acid [59,60,61,62,63,64,65]. Cyanidin- 3-O-saccharide was also reported from stems and leaves [66]. A comparative study of presence of these antioxidant constituents in blackberries is given below (Table 10). Fruits exhibit different antioxidant capacity due to variations in vitamin C and E contents, phenolic, flavonoid and anthocyanin contents, solvents used for extraction and method used to assess antioxidant activity. All these factors make it difficult to announce a definite antioxidant potential of fruits however it helps to get an idea about average antioxidant activity of fruits.

**Table 10:** Total polyphenols, total anthocyanins and ascorbic acid in blackberry.

Number of Cultivars	Total Polyphenols (mg/100 g)	Total Anthocyanins (mg/100 g)	Ascorbic Acid (mg/100 g)	Reference
4	2030	134–152	15.22	[36]
7	289.3	88.7	12.9	[67]
27	460	141	NR	[37]
2	417–555	110–122	NR	[56]
6	NR	NR	14.9	[68]
3	226	152.8	NR	[69]
1 (Chester)	361	NR	NR	[70]
5	320	80	20.4	[48]
4	07.5	115	NR	[71]
2	NR	NR	6	[72]

NR = Not Reported.

### 3.3. Carotenoids

Carotenoids are an important group of fat-soluble natural pigments and are believed to possess various immunity-boosting properties and health promoting effects. Marinova and Ribarova isolated lutein, β-cryptoxanthin, lycopene, zeaxanthin, β-carotene and α-carotene from *R. fruticosus* fruit [73]. Rutz *et al.*, investigated effect of maturity on lutein, zeaxanthin and β-carotene contents in pulp of blackberry fruit (Table 11). It was observed that carotene contents decreased with maturity stage of fruit [74].

**Table 11:** Carotenoid contents (μg/g) of blackberries (cv. Tupy) at different maturity stages [74].

Maturity Stage	Lutein + Zeaxanthin	*β*-Carotene
Immature	0.66	0.400
Intermediate	0.235	0.078
Mature	0.00	0.162

β-carotene has potential of transformation to vitamin A, thereby imparting an important nutritional role of acting as antioxidant to berry fruit.

### 3.4. Aromatic Compounds

Aromatic compounds are always present in plants as byproducts. Some important aromatic compounds isolated from the fruit of *R. fruticosus* are furans, 5-hydroxymethylfurfural and 2, 3-dihydro-3, 5-dihydroxy-6-methyl-4*H*-pyran-4-one. The aroma of blackberry fruit is due to presence of 5-hydroxymethylfurfural [75].

### 3.5. Triterpene Acids

**Figure 1:**
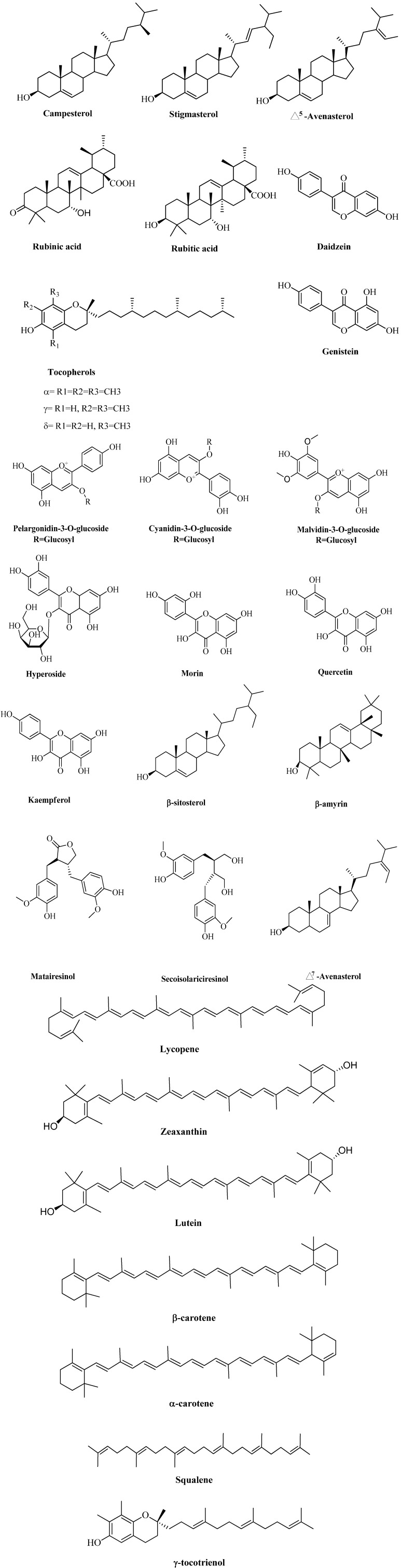
Phytochemicals isolated from *R. fruticosus*.

Triterpenoids are polycyclic compounds that are derived from linear hydrocarbon squalene and exert various biological activities due to their unique structure. Triterpene acid such as rubutic acid and rubinic acid were isolated from the leaves of *R. fruticosus*. 2-α-Hydroxyursolic acid and β-amyrin were also reported [76,77]. Figure 1 reports some of the main constituents found in *R. fruticosus.*

## 4. Traditional Uses

Fruits and fruit bearing plants are believed to possess various health-promoting effects and immunity-boosting properties since long ago. Romans treated various diseases through the use of tea prepared from its leaves [20]. *R. fruticosus* is known as food form about 8,000 years and as a medicinal plant soon after the Ice Age [77]. Hippocrates recommended blackberry stems and leaves soaked in white wine to relieve difficulties in childbirth and as an astringent poultice on wounds [78]. Externally it is used as a gargle to treat gum inflammations, sore throats and mouth ulcers [79,80]. Decoction of leaves is used as a gargle or mouthwash and also used to treat thrush [17]. The fruit juice is used to treat asthma [7]. The leaves of the plant are also used in various respiratory problems [81]. Blackberry juice is recommended in colitis while tea made from its roots is used for relief in labor pain. Poultice is leaves are applied in skin ulcers. The fruit and juice is recommended in anemia. *R. fruticosus* leaves or maceration of the tops in sunlight is claimed as cicatrizing agent [82]. A methanol extract of the aerial parts has been used for wound healing, as an antiseptic and a disinfectant and to treat cough [83,84]. *R. fruticosus* cures skin wounds in cattle [85]. A decoction of the twig tops soothes menstruations and also is used to treat diarrhoea. Its leaves are chewed to strengthen the gums and to cure thrush. Leaves are wrapped to stop fungal infection and abscesses on skin [86]. * R. fruticosus* jams, prepared without sugar, is prescribed to cure throat ailments in children and as an anti-diarrhea [87].The root-bark and the leaves are depurative, strongly astringent, tonic, vulnerary and diuretic. It is used as an excellent remedy against diarrhoea, dysentery, cystitis and haemorrhoids [7,86,87].

## 5. Pharmacological Actions

Fruits, vegetables, herbs and spices have been used since long to cure various human ailments besides their nutritional importance. This curative potential has been ascribed to various bioactive constituents and antioxidant components present in them and their synergistic effects. The most important activities of blackberry are anti-microbial, antioxidant, anti-inflammatory and anti-cancer. Several factors such as cultivar, agroclimatological conditions, level of ripening and processing method affect the profile and intensity of these pharmacological activities. Most activities performed are on crude extracts without sufficient information on preparation and standardization of extracts so many times results are non-reproducible. Most of pharmacological activities can be linked to various phenolic compounds which help in scavenging free radicals which are root cause of various pathological and metabolic disorders. Although many traditional uses have been verified, however *in vitro* as well as *in vivo* pre-clinical and clinical studies are necessary to assess their safety and efficacy.

### 5.1. Antimicrobial Activity

Riaz and coworkers studied the possible antibacterial activity of the methanol extracts from various parts of the plant against eight bacterial strains (*Salmonella typhi*, *Escherichia coli*, *Streptococcus aureus*, *Micrococcus luteus*, *Proteus mirabilis*, *Bacillus subtilis Citrobacteri sp.*, *Pseudomonas aeruginosa*). All extracts were found to inhibit growth of bacteria. The order of potency on minimum inhibitory concentration was stem > root > leaves > fruit. The same authors also screened the methanol extracts for their antifungal potential against nine pathogenic fungal strains (*Yersinia aldovae*, *Aspergillus parasiticus*, *Candida albicans*, *Aspergillus niger*, *Aspergillus effusus*, *Macrophomina phaseolina*, *Fusarium solani*, *Trichophyton rubrum, Saccharomyces cerevisiae*) without recording any biological activity [88]. Blackberry juice inhibited the growth of *Bacillus cereus*, *Bacillus subtilis*, *Streptococcus marcescens* and *Escherichia coli* from 50% to 75%. A methanol extract of aerial parts of *R. fruticosus* inhibited *Mycobacterium tuberculosis* with MIC of 1 mg/mL in agar dilution test [89]. Fruit cordials were reported to be bacteriostatic [90]. Abachi *et al.*, reported that MIC values of aqueous and ethanolic extracts against *Helicobacter pylori* were 400 and 450 µg/mL while zone of inhibitions were 8 and 7.3 mm for same extracts respectively [91]. Radovanović *et al.*, reported that blackberry extracts exhibited strong antioxidant potential against Gram (−) bacteria *S. enteritidis* ATCC13076and against Gram (−) bacteria *S. aureus* ATCC 6538, while weak to moderate activity was observed against *Clostridium perfringens* ATCC19404, *Bacillus subtilis* ATCC 6633, *Listeria innocua* ATCC33090, *Sarcina lutea* ATCC9341, *Micrococcus flavus* ATCC40240 and against gram negative bacteria like *Escerichia coli* ATCC25922, *Pseudomonas aeruginosa* ATCC9027, *Shigella sonnei* ATCC25931, *Klbsiella pneumonia* ATCC 10031 and *Proteus vulgaris* ATCC 8427 [37]. Yang *et al.*, reported that juice of fruits of *R. fruticosus* had strong antimicrobial potential against food borne pathogens like *Listeria monocytogenes*, *Salmonella Typhimurium*, *Escherichia coli*, *Lactobacillus casei*, *Lactobacillus plantarum* and *Lactobacillus rhamnosus*. The results suggest potential use of juice as a preservative in food processing industries [92]. Salaheen *et al.*, investigated the effect of extracts of blackberry pomace on growth and pathogenicity of *Campylobacter jejuni*. The extracts decreased the growth, swimming and swarming motility of *C*. *jejuni* and changed cell-surface hydrophobicity and auto-aggregation of these bacteria. The results indicate potential use of pomace extracts to reduce colonization level of *C*. *jejuni* in poultry and in controlling growth of pathogens in meat and meat products [93].

### 5.2. Antioxidant and Anticancer Activity

There is no standard method of preparation of extract of a plant part and its antioxidant analysis and it has led to diverse rather confusing reports when comparing the antioxidant potential of extracts of the same parts of the same plant, even from same regions with similar agro-geo-climatoligical conditions. Since different protocols for assessment of antioxidant capacity are based on different mechanisms, scientists therefore use a battery of assays when analyzing the antioxidant potential of any plant extract. Blackberries are a rich source of natural antioxidants as they contain high levels of phenols, flavonols and anthocyanins and are therefore well-reputed scavengers and inhibitors of free radicals [68]. Anthocyanins of blackberry are predominantly cyanidin based in non-acylated form. An ethanol extract of leaves was reported for its strong antioxidant potential [94]. Due to its antioxidant activity, blackberry exhibited chemopreventive effects in rats [38]. The antioxidant activity of fruit was also evaluated using ORAC method [68]. Blackberry extracts effectively suppressed the production of intracellular peroxyl free radicals induced by AAPH in human intestinal cell line (INT-407 cells) and this effect was concentration dependent. The suppression of intracellular oxidation by blackberry extract occurred at concentrations that were not toxic to the INT-407 cell line [95]. Blackberry powders were mixed with a synthetic diet (AIN-76), at 5% to 10% concentrations and fed to rats (Fischer 344) before, during and after treatment with the esophageal carcinogen N-nitrosomethylbenzylamine. At 25 week of experiment, all berry types inhibited the number of esophageal tumors (papillomas) in NMBA-treated animals by 24%–56% as compared to controls. This inhibition was associated with decrease in the formation of the NMBA-induced O-6-methylguanine adduct in esophageal DNA, indicating that the berries influenced the metabolism of NMBA leading to reduced DNA damage and thus preventing esophageal cancer in rats [38].

Cyanidin-3-O-glucoside isolated from blackberry possesses strong antioxidant activity and inhibited neoplastic transformation, metastasis, neoplastic cell migration and invasion, activation of tumor cell markers (NF-αB, AP-I, COX-2, TNF-α and MAPK), activation of cell migration markers (JNK, p38, and ERK), and induces apoptosis in neoplastic cell (HL-60 cells) [96].

Halvorsen and coworkers investigated the total antioxidant capacity of cultivated *R. fruticosus* collected at three different locations. The wild blueberry, wild blackberry, crowberry, sour cherry, black currant, wild strawberry, cultivated blackberry and cowberry/cranberry contained very high amount of total antioxidant concentration (5.03 to 9.17 mmol/100 g) [97]. Presence of anthocyanin in general and cyanidin-3-glucoside in particular in blackberry is the source of antioxidant capacity to repress both peroxyl-radical induced chemically and intracellular oxidation [95]. All conventional anticancer treatments like chemotherapy, surgery and radiotherapy have some side-effects. So scientists are looking for alternative anti-cancer remedies. Apoptosis of cancer cell is a unique target for chemoprevention study. A blackberry extract induced apoptosis in human leukemia HL-60 cells [98]. Some blackberry extracts (Hull Thornless, Chester Thornless, Triple Crown) induced apoptosis in human leukemia cells (HL-60) in a dose-dependent manner. Induction of apoptosis may be due to presence of various components in the extract; however the possible role of antioxidant potential of the extract may not be neglected in enhancement of cancer cells apoptosis. This indicates that there is a significant relationship between antioxidant activity, antioxidant content and anticancer activity in blackberries [99].

Wang and coworkers found that the pre-harvest application of methyl jasmonate (MJ) increased blackberry fruit quality significantly. MJ treated blackberries had low titratable acid content and high soluble solids as compared to untreated fruits. MJ treatment also significantly increased flavonoid contents, the antioxidant capacity and the inhibition of proliferation of cell lines (A549, HL-60) and also induced apoptosis in cell lines (HL-60) [100]. Tate and coworkers studied eight varieties of *R. fruticosus* (Arapaho, Chickasaw, Hull, Chester, Choctaw, Navajo, Kiowa, Triple Crown) to determine the inhibitory effect on UV-C–induced mutagenesis in *Salmonella typhimurium* TA100. Chester and Navajo varieties showed significant suppression of mutagenesis [101].

Intake of blackberry juice (BJs) prepared in water (BJW) and defatted milk (BJM) affects the plasma antioxidant power and non-enzymatic and enzymatic antioxidants. Ascorbic acid content increased significantly in plasma after intake of both BJs. However α-tocopherol and plasma urate were not affected. The plasma antioxidant capacity increased only after consumption of BJW. Plasma antioxidant capacity showed a positive correlation with ascorbic acid and a negative correlation with urate level. However no correlation was found among antioxidant capacity and total cyanidin or total ellagic acid contents. Intake of blackberry juice also increased plasma catalase level. A significant decrease in the urinary antioxidant capacity was observed [67]. Antioxidant profile of various cultivars is given in Table 12 [49]. Since synergetic effects exist between various bioactive compounds, so antioxidant capacity may be higher than measured for individual bioactive constituent, and therefore aggregate antioxidant potential of fruits should be measured instead of individual bioactive compounds.

**Table 12:** Phenolic, anthocyanin and ascorbic acid contents and DPPH radical scavenging activity of blackberry fruits [49].

Cultivar	Total Polyphenols (mg/100 g)	Total Anthocyanins (mg/100 g)	Ascorbic Acid (mg/100 g)	EC_50_ (mg)
Thornless Boy Sembes	329.1	126.9	12.5	5.2
Smoothstem	289	86.8	12.4	4.6
Black Diamond	307.4	119.3	13.1	5.7
Darrow	192.8	67.4	12.9	5.7
Hull Thornless	236.7	69.1	13.0	6.2
Chester	351.7	76.2	13.0	7.6
Black Satin	317.3	75.1	13.1	9.5
Means	289.3	88.7	12.9	6.4

Huang *et al.*, reported that blackberry extracts exhibited a strong DPPH scavenging activity (95.37%) at 2 mg/mL. Antioxidant activity observed was TEAC was 11.48 mmol Trolox/100 g DW, EC50 of DPPH was 0.44 mg/mL, TAC was 3.99 mg catechin/g DW,TFC was 11.83 mg rutin/g DW, TPC were v5.58 mg gallic acid/g DW. Phenolic acids, flavonoids and tannins detected were gallic acid; gallocatechin; protocatechuic acid; epigallocatechin; catechin;7, *p*-hydroxybenzoic acid; caffeic acid; malvidin-3-glucoside; *p*-coumaric acid; catechin gallate; cyanidin; ellagic acid; quercetrin (quercetin-3-rhamnoside); cinnamic acid and luteolin [102].

Samec *et al.*, investigated effect of temperature and time on blackberry fruits (Table 13). Storage of blackberry fruits at refrigerator temperature helped in preservation of fruit qualities by 1.6 to 5.5 fold as compared to at room temperature. Storage at 25 °C led to spoilage of analyzed fruits while storage at 4 °C did not adversely affect phytochemicals in analyzed fruits [103].

**Table 13:** Antioxidant components of blackberry fruits stored at 25 °C and 4 °C [103].

Days	Total Phenol Content (mg GAE/100 g FW)		Total Flavonoid Content (mg CE/100 g FW)		Total Anthocyanin Content (mg CGE/100 g FW)	
	25 °C	4 °C	25 °C	4 °C	25 °C	4 °C
0	364.24		66.13		121.82	
2	301.33	347.39	68.27	75.20	117.79	134.67
4	391.76	371.39	69.90	63.89	141.37	145.45
9		391.27		65.20		163.90
14		379.88		73.77		144.22

Stajčić *et al.*, reported chemical composition, total phenolic, flavonoid and monomeric anthocyanin contents as well as antioxidant activity two blackberry cultivars, *i.e.*, Čačanska bestrna and Thornfree (Table 14) [31].

**Table 14:** Total phenolic, flavonoid and monomeric anthocyanin contents and antioxidant activity two blackberry cultivars [31].

Parameter	Čačanska Bestrna	Thornfree
Total Phenolic Contents (mg GAE/100 g FW)	235.09	270.22
Total Flavonoind contents (mg RE/100 g FW)	143.33	172.95
Total Monomeric Anthocyanin Contents (mg CGE/100 g FW)	50.95	102.31
DPPH radical Scavenging Activity		
EC_50_ (mg FW/mL)	0.8188	0.6691
EC_50_ (mg extract/mL)	0.0616	0.0646

Radovanović *et al.*, also investigated antioxidant potential of blackberry fruits (Table 15). Phenolic acids identified were galic acid, caftaric acid, siringic acid, ferulic acid, while flavonoids detected were catechin, epicatechin, quercetin and quercitin-3-glycoside and rutin. All extracts showed high scavenging effect on DPPH radical with IC_50_ values ranging from 22.19 to 31.18 mL/g [40].

**Table 15:** Antioxidant potential of blackberry fruits [40].

Antioxidant Potential	Contents
Total phenols(mg GAE/kg)	7838.26
Total tartaric esters(mg CAEb/kg)	291.91
Total flavonols (mg QEc/ kg)	647.68
Radical scavenging activity(ml/g)	31.18

Ştefănuţ *et al.*, reported the anthocyanins, phenolics and antioxidant activity of fresh fruit of blackberry as 1,343 mg/L, 3,284 mg GAE/L and 17.3 (μM TE/gFM) respectively [39]. Percentage compositions of anthocyanins detected are reported in Table 16 [39].

**Table 16:** Anthocyanin contents of acidified ethanol extract of blackberry [39].

Anthocyanin Type	% of Total Anthocyanins
Cyanidin-3-sambubioside	0.84
Cyanidin-3-glucoside	90.72
Cyanidin-3-xyloside	3.44
Cyanidin-3-malonylglucoside	2.97
Cyanidin-3-dioxalylglucoside	2.04

Najda and Labuda reported total phenolic contents, anthocyanin contents and flavonoid contents of fresh fruits which were 101,947, 38,021 and 4,291 per 100 gram of fruits. Values for antioxidant activity (µMTE/g of fresh fruits) were 1,293, 971 and 517 respectively for DPPH, FRAP and ABTS [104]. Salehi *et al.*, determined effect of solvent on phenolic contents and antiradical activity of blackberry extracts (Table 17). Methanolic and *n*-hexane extracts contained highest and lowest amounts of phenolic contents respectively. Same trend was observed for DPPH radical scavenging assay [105].

**Table 17:** Total phenolic content and antiradical activity of blackberry extracts [104].

Extracts	Total Phenolic Content (mg GAE/g of Extract)	DPPH Radical Scavenging Activity (IC_50_ μg/mL)
*n*-Hexane	12	76.5
Dichloromethane	8.9	30.1
Chloroform	27	54.8
Ethylacetate	77.9	35.5
Methanol	79.1	15.2

Ivanovic *et al.*, studied effect of sonication time and temperature on yield, anthocyanin (cyanidin) contents and antioxidant potential of ultrasound-assisted extracts of blackberry. It was observed that increase of sonication time as well as temperature increased yield, anthocyanin contents as well as antioxidant potential of extracts. The results suggest use of ultrasound-assisted extraction technique for better isolation of anthocyanins from blackberry extracts [100]. Total phenolic contents and antioxidant activity of liqueurs made from different fruits was comparatively measured with regard to storage temperature and time (Table 18). In blackberry liqueur, the phenolic compounds, flavonols and anthocyanins decreased during storage. It is well-known that food commodities and plant parts like fruits and seeds undergo transformations during storage. Contents and composition of phenolic compounds present in them also change with the passage of time depending upon storage conditions. Anthocyanins are degraded because they are prone to oxidation and this process is sped up in the presence of vitamin C or its products. Similarly degradation process of phenolic compounds is initiated by various enzymes present in liqueur [106].

**Table 18:** Phenolic compound contents in liqueurs made from blackberry fruit at various time and storage intervals [106].

Month	Anthocyanin (mg cy-3-glu/100 mL)				Flavonols (mg Quercetin/100 mL)				Sum of Phenolic compounds (mg/100 mL)			
	15 ns	15 s	30 ns	30 s	15 ns	15 s	30 ns	30 s	15 ns	15 s	30 ns	30 s
0	26.6	22.4	22.1	22.6	1.9	1.6	1.4	1.5	37.4	33.4	33.0	33.6
3	14.7	15.4	0.2	0.4	0.9	1.0	0.0	0.0	23.6	26.1	8.8	10.6
6	8.8	9.7	0.0	0.0	0.4	0.6	0.0	0.0	16.6	19.0	8.8	10.6

15 ns-liqueurs without sugar stored in 15 C; 15 s-liqueurs with sugar stored in 15 C; 30 ns-liqueurs without sugar stored in 30 C; 30 s-liqueurs with sugar stored in 30 C.

Saponjac *et al.*,investigared anthocyanin contents and biological activities of two blackberry cultivars Thornfree (BT) and Cacanska bsetrna (BC). Cyanidin-3-O-glucoside was present in highest concentration being 1397.7 mg/Kg and 1360.6 mg/Kg in BT and BC respectively. Antioxidant activity determined via ABTS assay indicated EC50 of 0.007 and 0.06 g/L respectively for BC and BT respectively [107].

### 5.3. Anti-Inflammatory Activity

There is convincing evidence that increasing consumption of fruits reduces risk of inflammation. Fruit were found to be anti-inflammatory in murine model *in vivo*, with anthocyanins being responsible for this activity [108]. A water extract of fruits showed stronger anti-inflammatory activity even from aspirin by inhibiting hyaluronidase enzyme *in vitro* [109] thereby confirming traditional use of fruits as anti-inflammatory remedy. In another study inhibition of hyaluronidase enzyme was linked to GOD-type tannin [110].

An herbal composition for modulating cytokines in the regulation of inflammatory or immune diseases includes a blackberry extract [111]. Cyanidin-3-O-glucoside present in blackberry extract suppresses NO production which leads to anti- inflammatory effects. The mechanism of this inhibition may be due to an action on the expression/activity of the enzyme. Especially, the protein expression was inhibited by the attenuation of NF-κB and/or MAPK activation. The NF-κB activation is managed by mitogen-activated protein kinases (MAPK) [112]. It is used practically in the prevention and treatment of immune, inflammatory and metabolic diseases [113]. Sangiovanni *et al.*, investigated effects of allagitannin enriched extracts (ETs) of *R. fruticossus* for the control of gastric inflammation by *in vitro* and *in vivo* models. ETs inhibited TNFα-induced NF-κB driven transcription (IC50: 0.67–1.73 mg/mL) and IL-8 secretion (IC50: 0.7–4 mg/mL). Major ETs detected were sanguiin H-6 and lambertianin C which when tested decreased ulcer index by 88% and 75% respectively. The results confirm the protective effects of ETs in gastric inflammation [114].

### 5.4. Antidiabetic Activity

Diabetes mellitus (DM) is an endocrine and metabolic disorder characterized by dyslipidemia, hyperglycemia and protein metabolism that result from malfunction in regulating either insulin secretion or insulin action. Persons suffering from DM are more prone to risk of coronary heart diseases and therosclerosis. Despite the availability of modern hypoglycemic agents, ideal treatment of diabetes is still to be achieved, so scientists are searching for treatments from natural sources for diabetes mellitus. An aqueous tea prepared from balckberry fruit was evaluated by an *in vitro* glucose diffusion model but no anti-diabetic effect was recorded [115]. The water and butanol fractions of a *R. fruticosus* leaves 70% alcoholic extract were active in the treatment and prevention of noninsulin dependent diabetes. Water and butanol extracts from leaves of *R. fruticosus* were reported to be active in non-insulin dependent diabetes [116]. An aqueous extract of leaves was investigated for its possible anti-diabetic activity in rats. The hypoglycaemic effect demonstrated in normal rats indicates that it is active because counter-regulatory mechanisms cannot normalize rapidly blood glucose levels [117]. Chromium (Cr^3+^) and zinc (Zn^2+^) supplementation alleviates hyperglycemia and tea made from *R. fruticosus* leaves decreased diabetic symptoms associated with these metals dependent diabetes [118]. The leaves of *R. fruticosus* are advised practically to manage diabetes mellitus. Studies in streptozotocin (STZ)-diabetic mice have evaluated the anti-hyperglycaemic efficacy of RF previously as a dietary supplement. Blackberry fruit was found to exhibit no effect on glucose homeostasis in mice [9]. Leaves at daily administration of 5 g/kg of the infusion decreased 50% glucose-induced hyperglycemia in alloxan-diabetic rabbits [119,120]. Ştefănuţ reported that administration of blackberry extracts to diabetic rats in drinking water for 5 weeks significantly decreased glucose level from 360 to 270 mg/dL [39].

The general accepted therapeutic strategy for control of postprandial hyperglycemia is by inhibition of α-glucosidase and α-amylase enzymes. This leads to significant delay of carbohydrate breakdown to monosccharides. Salehi *et al.*, reported that *n*-hexane and chloroform extract of blackberry exhibited IC_50_ value of 0.5 and 6.2 in α-glucosidase inhibition activity while α-amylase inhibition potential of *n*-hexane and methanol extract was 53.7 thus indicating that extract may be used as potential anti-diabetic remedy [104]. Pressed residue of two blackberry cultivars Thornfree and Cacanska bsetrna exhibited stronger α-glucosidase inhibitory activity even at the lowest concentration, *i.e.*, 0.02 mg/mL, while complete inhibition was achieved at 0.63–2.50 mg/mL [107]. Collectively, the inhibition of intestinal α-glucosidase and pancreatic α-amylase activities as well as rich profile of antioxidant bioactive constituents indicate berry fruit as a promising dietic therapy for DM. Controlled clinical trials, however, are desirable for well-characterized and standardized blackberry extracts to corroborate its beneficial effects in diabetic patients. Similarly, traditional claimed use of its fruit to control hypertension and obesity should also be investigated in future studies.

### 5.5. Antiviral Activity

Globally viral diseases are increasing and simultaneously research to find new antiviral agents that are non-toxic and safe for human consumption is also increasing. The berry fruits are an ideal candidate for this search as these are non-toxic and may be recommended for human trials at lower costs. *R. fruticosus* is used in the treatment of influenza in combination with other medicinal plants [121] the role to control influenza virus may to the presence of polyphenols [122]. Antiviral activity data shows that very little work has been done on this aspect.

### 5.6. Neuropharmacological Activity

Our research group evaluated various activities on mice which are grouped as neuropharmacological activities.*R. fruticosus* L. fruit, leaves, root, and stem methanolic extracts were administered to mice at doses of 100, 300, and 500 mg/kg. The order of CNS depressant effect for various parts was fruit > root > leaves > stem. All extracts were found to be anxiolytic in nature, while no muscle relaxing activity or sedative effect was observed. The order of central nervous system (CNS) depressant effect for various parts of *R. fruticosus* was fruit > root > leaves > stem [123].

### 5.7. Toxicity Studies and Smooth Muscle Activities

Ali *et al.*, reported that LD_50_ of acute toxicity studies of crude methanolic extract of blackberry fruits was 887.75 ± 9.22 mg/mL while CC_50_ of same extract was 13.28 ± 2.47 μg/mL in brine shrimp cytotoxic studies. Excellent anthelmintic activity was exhibited by 20 mg/mL of extract against *Raillientina spiralis* and *Ascaridia galli* which was 1.37 times higher than albendazole. The extract although toxic is safe at 100 mg/kg. EC_50_ for spontaneous relaxant activity and for 80 mM KCl-induced contractions was 7.96 ± 0.1 and 6.45 ± 0.29 mg/mL respectively. The extract relaxed the spontaneous contractions in a concentration dependent manner on jejunum preparations. The results indicated that smooth muscle activity was mediated via inhibition of voltage gated channels [124].

### 5.8. Nutraceutical Usage

Blackberry juices, prepared with defatted milk and water, increased the ascorbic acid content in the plasma [27]. Health granules and health beverages are prepared from *R. fruticosus* and other plants used as dietary supplement and as immunity enhancer [125,126].

### 5.9. Miscellaneous Actions and Patents

Blackberry extract exhibited strong inhibitory action against monoamine oxidase B (MAO-B) and the inhibitory concentration, IC_50_, was found to be between 4 and 7 mg/mL [127]. Blackberry and its antioxidant components especially phenolics contribute positively to skin health by inhibiting the oxidative damage linked with the formation of wrinkles and other skin-disorders like hyperproliferation and skin dryness. It is used in cosmetic industry due to its specific scent and its antioxidant potential. It is frequently used in the formulation of skin care products, for facial cleansing, hair care products, to treat oily skin, acne as well as boils, skin eruptions and burns. Extracts of leaves are used for skin aging and deodorant composition [128,129,130,131,132].

A water extract of leaves is reported for its angiogenic properties [133]. Extracts of whole plant are used to prevent and cure inflammatory, immune and metabolic diseases and also as anti-influenza remedy [113,121]. The whole plant extract possesses diuretic and hypoazotemic activities [134].

An oral pharmaceutical formulation is prepared from *Gleditshcia triacanthos* powder, powdered leaves of *R. fructicosus*, pectin and corn starch and is used for the treatment of digestive disorders in calves and piglets [135]. A toothpaste containing *R. fruticosus* as active principle is used for dental caries, treating gums and cleaning teeth [136]. Leaves and fruits of *R. fruticosus* are consumed as traditional foodstuff in normal diet to maintain immune health [80,130]. Powdered fruit is also used as nutritional supplement [125].

### 5.10. Acute Toxicity

The lethality of water extract in mice was recorded for 1, 2, 3, 4 and 8 days after oral administration of various doses of *R. fruticosus*. The extract did not cause any death or significant changes in general behavior in mice at low and moderate doses (0–6 g/kg), but resulted in piloerection, rapid respiration and diuresis at higher doses (>6 g/kg).The LD_50_ value for the aqueous extract of leaves was 8.1 g/kg body weight [117].

## 6. Conclusions

*R. fruticosus* fruit is packed with numerous plant nutrients such as vitamins, minerals, anti-oxidants, and dietary fibers that are essential for human health and fitness. These compounds protect from cancer, aging, inflammation, and neurological diseases. With an increased awareness associated with potential health benefits of consuming fruits, efforts are being made to enhance fruit quality and color for consumers. It can be concluded that the wild growing blackberry fruits have a great future potential to meet nutritional demands of indigenous communities besides their therapeutic efficacy. More work is needed in identification, quantification and deciphering the bioactive constituents of fruit, seeds, flesh and peel of these berries and their impact on human health needs to be explored. Community-based trials should be conducted to validate its nutraceutical claims. The consumption of fruits, vegetables, spices, legumes and grains in Pakistan is insufficient and may be supplemented by indigenous cost-effective sources like berries. Although not a famous fruit for commercial production, it has a great production, expansion and consumption potential in Pakistan, if premium prices are paid and fruits are exploited economically. Even though various types of chemical compounds have been isolated and characterized, research reports on the bioactivity and the mechanism of action of the isolated compounds under *in vivo* conditions are limited. Additionally the effects of these compounds on other ailments like cancer, HIV, blood pressure, cardio-vascular disease and others, need to be investigated in detail.
